# Migration, Resilience, Vulnerability and Migrants’ Health

**DOI:** 10.3390/ijerph191811525

**Published:** 2022-09-13

**Authors:** Lillian Mwanri, Nelsensius Klau Fauk, William Mude, Hailay Abrha Gesesew

**Affiliations:** 1 Research Centre for Public Health Policy, Torrens University, Adelaide, SA 5000, Australia; 2 School of Health, Medical and Applied Sciences, Central Queensland University, Norman Gardens, QLD 4701, Australia; 3 College of Health Sciences, Mekelle University, Mekelle 1871, Tigray, Ethiopia

Migration has always been a feature of human populations, with people migrating and crisscrossing the globe for a wide range of reasons. During the 21st century [1], there have been substantial increases and changes in international migration and resettlement patterns due to factors including: people’s ability to travel, ease of communication and technology, civil unrest and conflicts, seeking opportunities for greater equality and freedom, and career progression and achievement. As a result of these factors, global populations have increased and integrated across settings, challenging the differentiation between types of migrants such as refugees and economic migrants.

As part of this exploration, up to 15 April 2022, a special topic of the *International Journal of Environmental Research and Public Health* (*IJERPH*) entitled “Migration, Resilience, Vulnerability and Migrants’ Health” was opened, and a dedicated team of scholars managed the editorial work as guest editors to facilitate the timely peer-review and publication of relevant manuscripts from multiple studies [2]. Between 20 February 2020 and 15 April 2022, a total of 44 manuscripts were submitted to the Special Issue, of which 14 were rejected and 29 published. A total of 128 authors from across the globe including Europe, Australia, China, and Malaysia contributed to the published articles. The published studies were conducted using different methodological approaches including mixed methods, qualitative, quantitative, and review studies. These studies involved participants whose migration involved both internal and international journeys, and they were both economic migrants and people with a refugee background. The published studies involved a wide range of population groups including men and women, children, young people, and people in different settings—such as aged care facilities, refugee camps, and in general community dwellings. By August 2022, the Special Issue had achieved 37,208 views.

In the Special Issue, a number of thematic areas were discussed including, but not limited to:

**A.**  **Health literacy and communication**—For example, Klingberg et al. [3] identified disparities in the use of emergency care between asylum seekers and Swiss nationals with non-urgent complaints, and Patel et al. [4] synthesized evidence on communication/interaction in the primary health care consultation setting with refugees or asylum seekers in western host countries.
**B.**  **Mental health and resilience**—For example, Hynek et al. [5] developed a system model of post-migration risk factors for mental health and their interactions, and Mwanri et al. [6] discussed how individual and community resilience factors supported the successful resettlement of Africans in Australia.
**C.**  **Sexual and reproductive health services**—For example, Loganathan et al. [7] explored the provision of sexual and reproductive health education, contraception, abortion, antenatal, and delivery, as well as the management of gender-based violence.
**D.**  **Identity and belongingness**—For example, Mude et al. [8] explored the identity and belonging of refugees in a host country.
**E.**  **Policy for disability among migrants in Europe**—For example, Martin-Cano et al. [9] critically reviewed the structure of social and professional intervention policies, at the international level, towards refugees with disabilities in Europe.

Conclusion: It is evident from these research activities that migrants, being internal or international and migrating for opportunities or as forced migrants (refugees), face a number of challenges, but opportunities do exist as well. Despite their vulnerability, especially for those migrating with a refugee background, through their resilience and adaptation to whatever adversity they face, they do survive and continue to contribute to their new place of residence.
